# Defects in Macrophage Reprogramming in Cancer Therapy: The Negative Impact of PD-L1/PD-1

**DOI:** 10.3389/fimmu.2021.690869

**Published:** 2021-06-23

**Authors:** Hao Cai, Yichi Zhang, Jian Wang, Jinyang Gu

**Affiliations:** Department of Transplantation, Xinhua Hospital Affiliated to Shanghai Jiao Tong University School of Medicine, Shanghai, China

**Keywords:** macrophage reprogramming, colony stimulating factors, programmed cell death ligand 1/programmed cell death 1, functional exhaustion, combination therapy

## Abstract

Classically activated M1 macrophages and alternatively activated M2 macrophages are two polarized subsets of macrophages at the extreme ends of a constructed continuum. In the field of cancer research, M2 macrophage reprogramming is defined as the repolarization of pro-tumoral M2 to anti-tumoral M1 macrophages. It is known that colony-stimulating factor 1 (CSF1)/CSF1 receptor (CSF1R) and CSF2/CSF2R signaling play important roles in macrophage polarization. Targeting CSF1/CSF1R for M2 macrophage reprogramming has been widely performed in clinical trials for cancer therapy. Other targets for M2 macrophage reprogramming include Toll-like receptor 7 (TLR7), TLR8, TLR9, CD40, histone deacetylase (HDAC), and PI3Kγ. Although macrophages are involved in innate and adaptive immune responses, M1 macrophages are less effective at phagocytosis and antigen presenting, which are required properties for the activation of T cells and eradication of cancer cells. Similar to T and dendritic cells, the “functionally exhausted” status might be attributed to the high expression of programmed death-ligand 1 (PD-L1) or programmed cell death protein 1 (PD-1). PD-L1 is expressed on both M1 and M2 macrophages. Macrophage reprogramming from M2 to M1 might increase the expression of PD-L1, which can be transcriptionally activated by STAT3. Macrophage reprogramming or PD-L1/PD-1 blockade alone is less effective in the treatment of most cancers. Since PD-L1/PD-1 blockade could make up for the defect in macrophage reprogramming, the combination of macrophage reprogramming and PD-L1/PD-1 blockade might be a novel treatment strategy for cancer therapy.

## Introduction

Macrophages exhibit a high degree of plasticity when exposed to various environmental stimuli. They are polarized to one of two opposite types *in vitro*, classically activated M1 macrophages that can be induced by lipopolysaccharide (LPS), interferon-γ (IFN-γ), or colony-stimulating factor 2 (CSF2, also known as granulocyte-macrophage colony-stimulating factor) or alternatively activated M2 macrophages that can be induced by interleukin 4 (IL4), IL13, or CSF1 (also known as macrophage colony-stimulating factor) (1–3). M2 macrophages are further categorized as M2a, M2b, M2c, and M2d cells upon stimulation of different M2 drivers (4). Generally, M1 macrophages exert an immune protective role *via* the secretion of pro-inflammatory cytokines, whereas M2 macrophages are characterized by anti-inflammatory properties, which contribute to tissue remodeling and tumor progression (3). Multiple co-stimulatory and antigen-presenting molecules are expressed on the cell membrane of antigen-presenting cells (APCs), including macrophages. When confronted by tumor antigens, macrophages engulf and present them to T cells to boost the anti-tumor immune reaction by acting synergistically with co-stimulatory molecules (1). However, the function of macrophages is more complex in the tumor microenvironment. Tumor-associated macrophages (TAMs) are thought to exhibit an M2-like phenotype, lose their antigen-presenting capacity as innate immune cells, and play a pro-tumoral role in the tumor microenvironment in a paracrine manner (5, 6). The phenotype of TAMs dynamically changes with the development and progression of tumors. At an early stage, macrophages harboring anti-tumor capacity are recruited to the tumor microenvironment; however, with tumor progression, these macrophages are “educated” by tumor-secreted cytokines to acquire an M2 phenotype (1). It is accepted that M1 and M2 are two extreme forms of polarization *in vitro*, and TAMs usually exhibit a mixed M1–M2 phenotype, and not a simple M1 or M2 only phenotype, *in vivo* (7–9).

Macrophage reprogramming, also called macrophage repolarization, is defined as the repolarization of differentiated macrophages from alternatively activated M2 phenotype to the classically activated M1 phenotype, and vice versa. Several methods have been developed to reprogram M2 macrophages, including use of targeted antibodies, small molecular inhibitors, and free or vector-delivering nucleic acids, among others. Although M2 macrophage reprogramming has been adopted in clinical trials, the treatment outcome remains uncertain. In this review, we aim to shed light on the defects in M2 macrophage reprogramming and provide better treatment strategies for cancer therapy.

## Macrophage Reprogramming Strategies

Molecular targets for M2 macrophage reprogramming include Toll-like receptor 7 (TLR7), TLR8, TLR9, CD40, histone deacetylase (HDAC), PI3Kγ, CSF1, and CSF1 receptor (CSF1R) (10). TLR agonists induce M1 polarization and exert an anti-tumor effect *via* the increased release of pro-inflammatory mediators. CD40 agonists increase the expression of co-stimulatory and antigen-presenting molecules on macrophages and the secretion of pro-inflammatory mediators, which enhances the T cell–dependent anti-tumor effect (10). TLR signaling and CD40 are known to be activated by IFN-γ (11, 12), which is a driver of M1 polarization. Both HDAC and PI3Kγ are involved in the M2 polarization of macrophages, providing intracellular targets for macrophage reprogramming. The inhibition of HDAC or PI3Kγ exerts an anti-tumor effect *via* the downregulation of M2 and upregulation of M1 molecules (10). As has been reported, PI3K is present downstream of CSF1R and is epigenetically activated during M2 polarization (13).

The CSF1/CSF1R axis is the most attractive target to reprogram M2 macrophages, and multiple agents have been developed for clinical practice, including small molecule inhibitors (PLX3397, BLZ945, ARRY-382, etc.) and neutralizing antibodies against CSF1 or CSF1R (10, 14). In the tumor microenvironment, tumor cell-derived CSF1 is enriched within peri-tumoral tissues and functions as a chemoattractant to recruit circulating monocytes, subsequently resulting in increased macrophage infiltration (15). CSF1R is a transmembrane receptor for CSF1 and IL34 with tyrosine kinase activity. Binding of CSF1 or IL34 induces the homodimerization of CSF1R and the activation of downstream MEK, PI3K, and PLC-γ2 signaling pathways, which are crucial for the proliferation and differentiation of macrophages (13). It was reported that CSF1/CSF1R blockade-based anti-tumor therapy could result in loss of macrophages in the tumor either by mitigating recruitment, TAMs survival and/or differentiation from monocytes (3). Ao et al. reported that CSF1R inhibitor PLX3397 suppressed tumor growth without depletion of TAMs infiltration in a mouse model of liver cancer (16). These discrepancies might be attributed to the heterogenicity of different tumor species and different CSF1/CSF1R blockade agents. It is accepted that activation of the CSF1R signaling pathway induces the M2 polarization of macrophages (14). In contrast with CSF1/CSF1R signaling that induces M2 polarization, the CSF2/CSF2R pathway induces M1 polarization of macrophages. CSF1R and CSF2R are constitutively expressed on the cell membrane of macrophages. Both CSF1/CSF1R and CSF2/CSF2R signaling pathways play important roles in macrophage reprogramming. Infiltrating macrophages are “educated” by tumor cell-derived CSF1 or IL34 to acquire an M2 phenotype, characterized by the high expression of CD163 or CD206 (17–19). After blockade of the CSF1R signaling pathway, macrophages are repolarized instead through the CSF2/CSF2R axis to acquire an M1 phenotype (16). CSF2 enhances the antigen presentation capacity of macrophages with increased expression of major histocompatibility complex-II (MHC-II) (20). In addition to inducing M1 polarization, CSF2 is also responsible for the development of dendritic cells (DCs) and granulocytes, which are also APCs that exert a similar anti-tumor effect as M1 macrophages (21, 22).

However, it has been reported that traditionally defined M1 macrophages with “anti-tumor properties” could also facilitate the metastasis of hepatocellular carcinoma in a paracrine manner (23). IL-1β released by M1 macrophages induces the expression of co-inhibitory molecules in tumor cells, which hampers the direct anti-tumor effect of cytotoxic T cells (24). Although these M1 macrophages are presented with high MHC-II expression, the process of phagocytosis and antigen presentation does not function as expected and needs further investigation. Therefore, after macrophage reprogramming from M2 to M1, the restoration of the phagocytic and antigen-presenting capacity of macrophages as innate immune cells should be taken into consideration to develop strategies for anti-tumor therapy.

## Independent Role of the PD-L1/PD-1 Axis in Macrophages

Programmed cell death-ligand 1 (PD-L1) was reported to be expressed on a variety of cells, including tumor cells and APCs (mainly DCs and macrophages), and acts as a ligand for programmed cell death protein 1 (PD-1), which is mainly expressed on T cells. The binding of PD-L1 on macrophages to PD-1 on T cells antagonizes the co-stimulating and antigen-presenting effect of macrophages on T cells, leading to T-cell anergy and tumor cell immune escape. In addition to being a ligand for PD-1, PD-L1 inhibits the proliferation and activation of macrophages by suppressing the mechanistic target of rapamycin signaling pathway in macrophages. In addition, PD-L1 induces an immunosuppressive phenotype and inhibits the antigen-presenting capacity by reducing the expression of co-stimulatory molecules in macrophages. PD-L1 blockade increases the production of co-stimulatory molecules (CD86 and MHC-II) and pro-inflammatory cytokines (tumor necrosis factor α [TNFα] and IL12), which comprises the phenotype and expression profile of M1 macrophages (25). However, transcriptomic analysis showed that in addition to increasing the expression of pro-inflammatory genes, CCL2, a key driver of macrophage recruitment and M2 polarization is also upregulated. It was reported that PD-L1 blockade reinvigorates T cells, which is accompanied by increased IFN-γ production, further driving the M1 polarization of macrophages (26). However, the direct impact of PD-L1 on macrophage polarization is uncertain. The correlation between PD-L1 expression and macrophage polarization is ambiguous. Multiple factors involved in both M1 and M2 polarization (IFN-γ, TNFα, LPS, IL4, IL6, IL10, IL13, etc.) increase the expression of PD-L1 on macrophages (27–31). Therefore, PD-L1 is not an exclusive biomarker of M1 or M2 macrophages.

Recently, PD-1 was also found to be expressed on TAMs but not on circulating monocytes or spleen macrophages. The tumor microenvironment might play a critical role in the expression of PD-1 in macrophages. PD-1 inhibits the phagocytosis of TAMs, which is an inherent attribute of APCs in innate immunity (32). Peritoneal macrophages with high PD-1 expression are dysfunctional with reduced bactericidal capacity (33). It has been reported that macrophages with high PD-1 expression are more likely to be the pro-tumoral M2 subtype with increased CD206 expression and reduced MHC-II expression (32). Rao et al. reported that anti-PD-1 therapy induces M1 polarization in macrophages and exerts an anti-tumor effect in the absence of CD8^+^ T cells (34). In this study, the authors concluded that combining macrophage reprogramming with anti-PD-1 therapy is unnecessary, because anti–PD-1 alone can eliminate PD-1-expressing microglia, thus driving M2 to M1 repolarization. However, this result should be interpreted with caution. Even after PD-1-expressing macrophages have been eliminated, M1 polarization of the remaining macrophages might not occur without extra stimulation or induction. Thus, the role of CSF2 or IFN-γ should be taken into consideration.

As reported, activation of the PD-L1/PD-1 axis represents a state of “functional exhaustion” in T cells and DCs (35–37). Exhausted T cells are initially functional when exposed to antigen but gradually become silent after persistent stimulation. The exhausted T cells are characterized by increased expression of inhibitory molecules and decreased secretion of effector cytokines, including IL2, IFN-γ, and TNFα. The inhibitory receptors expressed on exhausted T cells include PD-1, cytotoxic T-lymphocyte-associated protein 4 (CTLA4), T-cell immunoreceptor with Ig and ITIM domains (TIGIT), lymphocyte-activation gene 3 (LAG-3), B and T lymphocyte attenuator (BTLA), T-cell immunoglobulin and mucin-domain-containing protein 3 (TIM3), V domain Ig suppressor of T-cell activation (VISTA), and CD96, among others (38). Similarly, these macrophages with high PD-L1 or PD-1 expression could also be called “functionally exhausted.” PD-L1 or PD-1 expression hampers the anti-tumor effect of macrophages as innate and adaptive immune cells, which could be restored after PD-L1/PD-1 blockade.

## M2 to M1 Reprogramming is Associated With Increased PD-L1 Expression

Analysis of Gene Expression Omnibus data sets (GSE95404, 71253, 66805, 95405, 69607) by comparing the transcriptional differences of M1 (induced by CSF2 or LPS/IFN-γ) and M2 (induced by CSF1 or IL4) macrophages revealed that PD-L1 expression is significantly higher in M1 macrophages than in M2 macrophages. Antonios et al. showed that the expression of PD-L1 is also significantly higher in CD163^−^ cells than in CD163^+^ cells (39). A recent study identified an immune suppressor induced by CSF1, Siglec-15, which is negatively correlated with PD-L1 expression (40). CSF1R blockade increases the expression of PD-L1 in pancreatic ductal tumor cells and CD11b^+^Ly6C^high^ monocytes (41), but the underlying mechanism remains unknown. CSF2 plays an important role in directing M1 polarization after blockade of the CSF1/CSR1R signaling pathway, which is involved in M2 polarization. Shelby et al. showed a positive correlation between PD-L1 and CSF2 concentrations in gingival crevicular fluid of patients diagnosed with periodontitis (42). It has been reported that the expression of PD-L1 on APCs is transactivated by STAT3 (43). Tumor-derived CSF2 increases the expression of PD-L1 in granulocytes by activating the JAK/STAT3 signaling pathway, which inhibits the anti-tumor effect of T cells and contributes to the progression of gastric cancer (44) and hepatocellular carcinoma (45). In liver myeloid-derived suppressor cells, activation of the CSF2/JAK2/STAT3 pathway increases the expression of PD-L1, which facilitates the intrahepatic metastasis of liver neoplasm (46). It was also reported that CSF2 increases the secretion of CXCL8 in macrophages, which further induces the expression of PD-L1 on TAMs in an autocrine manner and inhibits the anti-tumor effect of CD8^+^ T cells (47). However, the underlying mechanism through which CSF2 increases PD-L1 expression on macrophages has not been fully elucidated.

**Figure 1:**
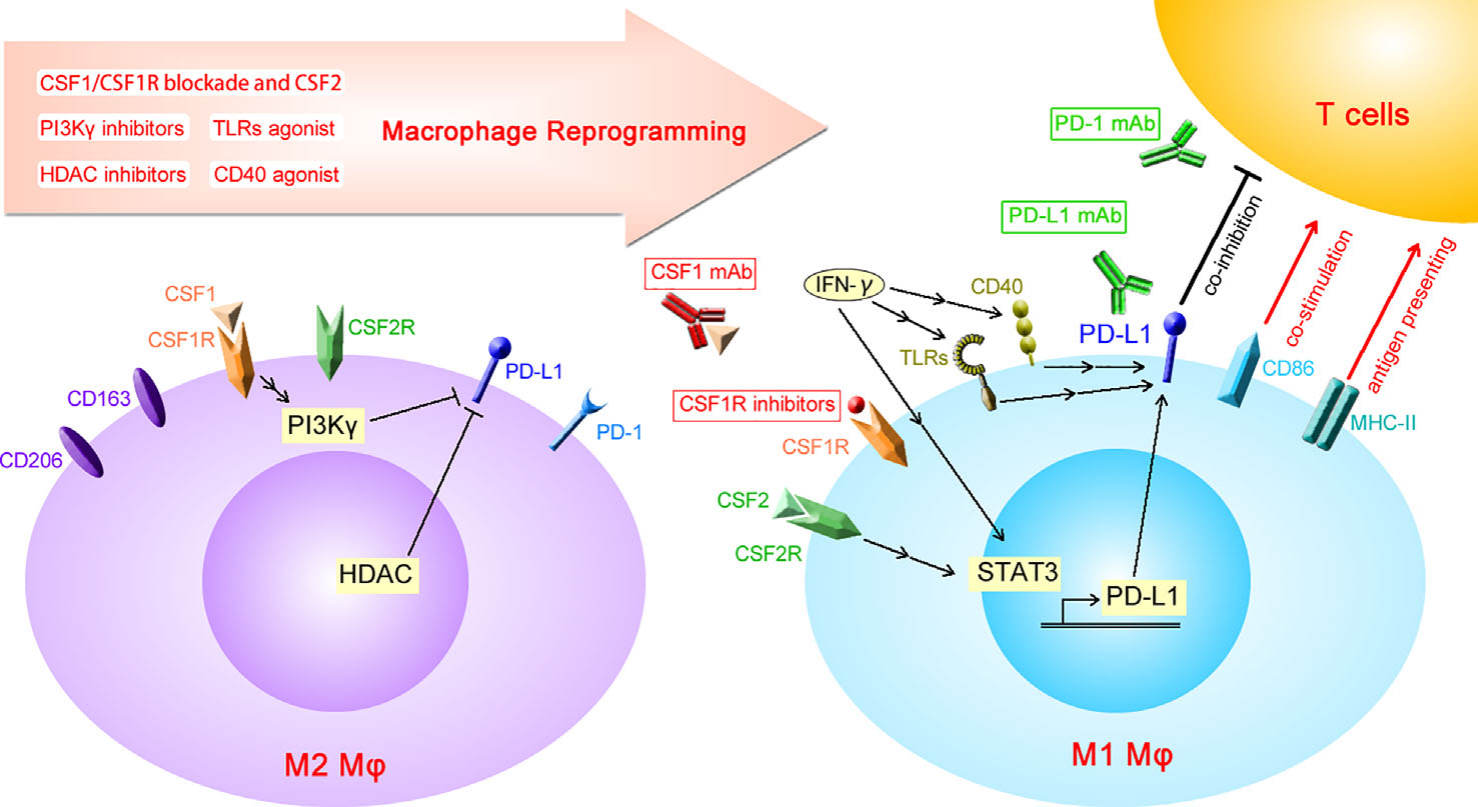
Diagrammatic sketch of macrophage reprogramming increasing the expression of PD-L1. PD-L1, programmed death-ligand 1; PD-1, programmed cell death protein 1; CSF1, colony-stimulating factor 1; CSF1R, CSF1 receptor; CSF2, colony-stimulating factor 2; CSF2R, CSF2 receptor; HDAC, histone deacetylase; IFN-γ, interferon-γ; TLRs, toll-like receptors; MHC-II, major histocompatibility complex-II; mAb, monoclonal antibody; Mφ, macrophages.

PD-L1 expression is known to increase in M1 macrophages when induced with CSF2, LPS, or IFN-γ. An *in vitro* study showed that phorbol-12-myristate 13-acetate-activated THP-1 macrophages exhibit higher PD-L1 expression after LPS stimulation (48). IFN-γ released by tumor-infiltrating T lymphocytes increases PD-L1 expression in macrophages, which in turn inhibits the anti-tumor effect of T cells, resulting in a state of “adaptive immune tolerance” (49, 50). Both IFN-γ- and LPS-induced PD-L1 expression on tumor-infiltrating macrophages is dependent on the activation of STAT3 (51, 52). Moreover, activation of the TLR4/ERK axis is responsible for LPS-induced PD-L1 expression (53). In addition to CSF1/CSF1R blockade, macrophage reprogramming using HDAC inhibitors or agonistic anti-CD40 antibodies increases the expression of PD-L1 on macrophages, which limits the anti-tumor effect of reprogrammed TAMs (54, 55). Although the impact of macrophage reprogramming targeting PI3Kγ on PD-L1 expression is unknown, it has been reported that PI3Kγ inhibition increases the expression of PD-L1 on myeloid-derived suppressor cells (56). This evidence shows that all of these macrophage reprogramming strategies have the same side effect of increased PD-L1 expression on macrophages (**Figure 1**). Although PD-1 is also reportedly found on macrophages, the impact of macrophage reprogramming on PD-1 expression has not yet been reported.

## Macrophage Reprogramming and PD-L1/PD-1 Blockade in Cancer Therapy

### Shortcomings of Single-Agent Anti-Tumor Strategies

In recent years, PD-L1/PD-1 monoclonal antibodies have gained widespread attention for cancer treatment. They show a strong anti-tumor effect in certain cancers such as Hodgkin’s disease and desmoplastic melanoma, among others. However, in most cancers, including non-small-cell lung carcinoma, gastroesophageal cancer, urinary neoplasm, and hepatocellular carcinoma, PD-L1/PD-1 blockade alone is only effective in a small proportion of patients (with objective response rates ranging from 15% to 25%) (57). Resistance to PD-L1/PD-1 blockade might be attributed to a lack of pre-existing T-cell infiltration in certain tumors. Another mechanism for ineffective anti-PD-L1/PD-1 monotherapy is the increased macrophage infiltration in the tumor microenvironment (58). Tumor-derived CSF1 induces the expression of granulin in macrophages, which impedes the infiltration of cytotoxic CD8^+^ T cells at the site of the tumor lesion, resulting in resistance to immune checkpoint therapy (59, 60). Therefore, therapies combining anti-PD-L1/PD-1 monoclonal antibodies have been adopted as a priority for clinical practice for selected cancers. For advanced hepatocellular carcinoma and clear cell renal cell carcinoma, anti-PD-L1/PD-1 single-agent therapy is only recommended as a second-line treatment option when first-line treatment fails (61, 62).

Even though macrophage reprogramming using CSF1R inhibitors or CSF1 antibodies has been achieved for anti-tumor therapy, their effectiveness is still uncertain in most cancers. The CSF1R inhibitor PLX3397 has shown significant anti-tumor effects in animal models (63–67), but its treatment efficacy in clinical settings remains unknown. PLX3397 is effective for tenosynovial giant cell tumors (68, 69) with a 39% overall response rate in a phase III clinical trial (69). However, the treatment outcome was rather disappointing for glioma (70). A poor objective response rate was reported for the CSF1R inhibitor ARRY-382 and CSF1R monoclonal antibody emactuzumab in treating solid tumors. Data on tumor control are unavailable for the CSF1 monoclonal antibody lacnotuzumab and CSF1R monoclonal antibodies cabiralizumab and LY3022855 (14). Similar to CSF1/CSF1R blockade, CSF2 induces M1 polarization by activating the CSF2R signaling pathway. Moreover, CSF2 is used to augment the recruitment and maturation of DCs in clinical trials for cancer therapy. However, CSF2 is typically not used as a single agent for macrophage reprogramming in clinical settings. Except for CSF1/CSF1R blockade and CSF2, other macrophage reprogramming agents including CD40 agonists, TLR agonists, HDAC inhibitors, and PI3Kγ inhibitors have been approved in clinical practice for the treatment of selected types of tumors (71–74). All of these macrophage reprogramming strategies are only effective in a small number of patients. As has been detailed in this review, the side effect of increased PD-L1 expression might explain the relatively poor anti-tumor effect of macrophage reprogramming. The anti-tumor nature of macrophages is inhibited by PD-L1, which could be rescued by PD-L1/PD-1 blockade.

Macrophages play a critical role in the activation of T cells (75). Activated CD8^+^ T cells mediate tumor cell killing directly, whereas activated CD4^+^ T cells exert anti-tumor effects indirectly by enhancing the cytotoxic effect of CD8^+^ T cells. The T cell–dependent anti-tumor response requires not only the blockade of co-inhibitory signals on T cells and APCs but also the restoration of co-stimulatory and antigen-presenting molecules on APCs. Macrophage reprogramming-induced PD-L1 expression provides a therapeutic target for PD-L1/PD-1 monoclonal antibodies. Moreover, PD-L1/PD-1 monoclonal antibodies could make up for the defect in macrophage reprogramming, thus improving the anti-tumor effectiveness of macrophage reprogramming. Roemer et al. reported that the expression of both MHC-II and PD-L1 is associated with a favorable outcome with PD-1 blockade (76). Bioinformatics analysis has shown that M1 macrophages are required for the efficacy of anti-PD-L1/PD-1 therapy (77), which implies better treatment outcomes by combining macrophage reprogramming and anti-PD-L1/PD-1 therapy compared to those of single-agent anti-tumor strategies. In this review, CSF1/CSF1R and CSF2/CSF2R-based macrophage reprogramming and its combination with PD-L1/PD-1 blockade is emphasized. The completed and ongoing clinical trials combining macrophage reprogramming and PD-L1/PD-1 blockade in cancer therapy are summarized in **Table 1**.

**Table 1:** Completed and ongoing clinical trials combining macrophage reprogramming and PD-L1/PD-1 blockade in cancer therapy.

Macrophage reprogramming		PD-L1/PD-1 blockade	Cancer types	Clinical phase	Status/outcomes	Clinical trial identifier
CSF1R inhibitors	PLX3397	Durvalumab	Metastatic/advanced pancreatic or colorectal cancers	I	Completed (no results)	NCT02777710
	SNDX-6352	Durvalumab	Unresectable intrahepatic cholangiocarcinoma	II	Recruiting	NCT04301778
	ARRY-382	Pembrolizumab	Advanced solid tumors	Ib/II	Completed (no results)	NCT02880371
	DCC-3014	Avelumab	Advanced or metastatic sarcomas	I	Recruiting	NCT04242238
	BLZ945	PDR001	Advanced solid tumors	I/II	Recruiting	NCT02829723
CSF1R mAb	Cabiralizumab	Nivolumab	Peripheral T-cell lymphoma	II	Active (no results)	NCT03927105
	LY3022855	Durvalumab	Advanced solid tumors	I	Completed (no results)	NCT02718911
	Emactuzumab	Atezolizumab	Solid cancers	I	Completed (no results)	NCT02323191
CSF1 mAb	PD-0360324	Avelumab	Advanced cancer	Ib/II	Recruiting	NCT02554812
	lacnotuzumab	PDR001	Solid tumors	I/II	Completed (no results)	NCT02807844
CSF2	Pexa-Vec (Thymidine Kinase- Deactivated Vaccinia Virus Plus GM-CSF)	Cemiplimab	Renal cell carcinoma	Ib/IIa	Recruiting	NCT03294083
CD40 agonist	Selicrelumab	Atezolizumab	Solid tumors	Ib	Completed (no results)	NCT02304393
	APX005M	Nivolumab	Non-small cell lung cancer or metastatic melanoma	I/II	Completed (no results)	NCT03123783
TLRs agonist	DV281	Approved anti-PD-1 inhibitor	Advanced non-small cell lung cancer	Ib	Completed (no results)	NCT03326752
	BO-112	Pembrolizumab	Melanoma	II	Recruiting	NCT04570332
	VTX-2337	Nivolumab	Squamous cell carcinoma of the head and neck	Ib	Recruiting	NCT03906526
	SBT6050	Pembrolizumab	HER2-positive solid tumors	I	Recruiting	NCT04460456
	CMP-001	Avelumab	Advanced cancer	Ib/II	Recruiting	NCT02554812
HDAC inhibitors	Entinostat	Nivolumab	Previously treated unresectable or metastatic cholangiocarcinoma and pancreatic adenocarcinoma	II	Active (no results)	NCT03250273
	Domatinostat	Avelumab	GI cancer	II	Recruiting	NCT03812796
	Entinostat	Pembrolizumab	Myelodysplastic syndrome	I	Active (no results)	NCT02936752
PI3Kγ inhibitors	Copanlisib	Nivolumab	Relapsed/refractory solid tumors with expansions in mismatch-repair proficient (MSS) colorectal cancer	I/II	Recruiting	NCT03711058
	Copanlisib	Nivolumab	Richter’s transformation or transformed indolent non-Hodgkin’s lymphoma	I	Recruiting	NCT03884998

### CSF1/CSF1R Blockade Combined With a PD-L1/PD-1 Monoclonal Antibody

A preclinical study showed that PLX3397 combined with a PD-1 monoclonal antibody enhances the anti-tumor effect of a DC vaccine in a mouse glioma model (39). Another CSF1R inhibitor, BLZ945, was also reported to synergize with PD-1/PD-L1–blocking antibodies for the treatment of murine neuroblastoma (78). However, in clinical practice, a lack of evidence on tumor control has limited the use of the combination of CSF1/CSF1R blockade and PD-L1/PD-1 monoclonal antibody to treat solid tumors. A phase II clinical trial using the combination of a CSF1R antibody (AMG820) and pembrolizumab has revealed an acceptable safety profile. However, the anti-tumor effect was insufficient for further evaluation, which might be because most recruited patients were resistant to the PD-1 antibody (79). Recently, mannose-modified macrophage-derived microparticles loaded with metformin have been developed to reprogram M2 to M1 macrophages, which can synergistically enhance anti-PD-1 therapy (80).

### CSF2 Combined With a PD-L1/PD-1 Monoclonal Antibody

A preclinical animal study has shown that anti-PD-1 therapy increases the anti-tumor effect of CSF2 (81). CSF2 is used as a single agent for treating melanoma and was shown to provide no survival benefits in a phase III clinical trial (82). Holi et al. reported that treatment with immune checkpoint inhibitor ipilimumab plus sargramostim (CSF2) showed longer survival (1-year overall survival: 68.9% *vs* 52.9%) and lower toxicity (grades 3–5 toxicity: 44.9% *vs* 58.3%) than ipilimumab alone in treating metastatic melanoma (83). In most cases, CSF2 is usually anchored to the tumor vaccine as an adjuvant. PD-L1/PD-1 blockade was shown to increase the anti-tumor effect of the anchored-CSF2 tumor vaccine (84–86). Tian et al. constructed a new type of tumor vaccine that produces both PD-1 antibody and CSF2, which has shown a promising anti-tumor effect (87). In gallbladder cancer, conventional chemotherapy supplemented with CSF2 and PD-L1 blockade was shown to decrease local cancer recurrence after surgery (88). Therefore, CSF2, in combination with a PD-L1/PD-1 monoclonal antibody, is superior to single-agent therapy.

## Conclusion

Macrophage reprogramming has been adopted in clinical trials for cancer therapy. Several reprogramming strategies have been developed by targeting TLR7, TLR8, TLR9, CD40, histone deacetylase (HDAC), PI3Kγ, CSF1, and CSF1R. The CSF1/CSF1R axis is the most attractive target to reprogram M2 macrophages in clinical trials. However, traditionally defined M1 macrophages with “anti-tumor properties” could also facilitate cancer progression, even with high expression of co-stimulatory and antigen-presenting molecules. The side effect of increased PD-L1 expression results in a “functionally exhausted” status in macrophages, which limits the anti-tumor effect of reprogrammed macrophages. PD-L1/PD-1 blockade could make up for the defect in macrophage reprogramming, providing a potentially promising treatment strategy by combining macrophage reprogramming with PD-L1/PD-1 monoclonal antibodies.

## Author Contributions

JG, HC, and YZ conceived the topic of this review article. HC, YZ, and JW searched reference articles and extracted key information for this review article. HC and YZ wrote this manuscript. All authors contributed to the article and approved the submitted version.

## Funding

This work was supported by grants from the National Natural Science Foundation of China (81772507, 82072646 to JG), Key Program of Development Center for Medical Science & Technology National Health Commission of China (NHC2018RWS01007 to JG), Shanghai Municipal Education Commission---Gaofeng Clinical Medicine Grant Support (20191910 to JG) and Clinical Research Plan of SHDC (no. SHDC2020CR3005A to JG).

## Conflict of Interest

The authors declare that the research was conducted in the absence of any commercial or financial relationships that could be construed as a potential conflict of interest.

## Abbreviations

CSF1, colony-stimulating factor 1; CSF1R, CSF1 receptor; DCs, dendritic cells; PD-1, programmed cell death protein 1; PD-L1, programmed cell death-ligand 1; APCs, antigen-presenting cells; IFN-γ, interferon-γ; IL, interleukin; LPS, lipopolysaccharide; TAMs, tumor-associated macrophages; HDAC, histone deacetylase; TLR7, Toll-like receptor 7; MHC-II, major histocompatibility complex-II.
